# CDC20 overexpression predicts a poor prognosis for patients with colorectal cancer

**DOI:** 10.1186/1479-5876-11-142

**Published:** 2013-06-10

**Authors:** Wen-jing Wu, Kai-shun Hu, De-shen Wang, Zhao-lei Zeng, Dong-sheng Zhang, Dong-liang Chen, Long Bai, Rui-hua Xu

**Affiliations:** 1 State Key Laboratory of Oncology in Southern China, Guangzhou 510060, China; 2 Department of Experimental Research, Sun Yat-sen University Cancer Center, Guangzhou 510060, China; 3 Department of Medical Oncology, Sun Yat-sen University Cancer Center, Guangzhou 510060, China; 4 Key Laboratory of malignant tumor gene regulation and target therapy of Guangdong Higher Education Institutes, Research Center of Medicine, Sun Yat-sen Memorial Hospital, Sun Yat-sen University, Guangzhou 510120, China

**Keywords:** CDC20, Prognosis, Colorectal cancer

## Abstract

**Background:**

The cell division cycle 20 homolog (CDC20) is an essential cofactor of the anaphase-promoting complex (APC/C). CDC20 overexpression has been detected in many types of human cancers; however, its clinical role in colorectal cancer remains unknown.

**Methods:**

Western blotting and immunohistochemistry were used to compare CDC20 expression in adjacent non-cancerous, cancerous and liver metastatic tissues as well as in colon cancer cell lines and normal colon epithelial cell lines. Additionally, the correlation of CDC20 expression with patient clinical parameters and its diagnostic value were statistically analyzed.

**Results:**

CDC20 was overexpressed in colon cancer cell lines/primary cancer tissues compared with normal colon epithelial cell lines/adjacent noncancerous tissue samples. Interestingly, CDC20 expression was further increased in metastatic liver tissues. CDC20 protein expression was significantly correlated with clinical stage (*P* = 0.008), N classification (*P* = 0.020), M classification (*P* = 0.013) and pathologic differentiation (*P* = 0.008). Patients with higher CDC20 expression had a shorter overall survival than those with lower CDC20 expression. Univariate and multivariate analyses indicated that CDC20 expression was an independent prognostic factor (*P* < 0.001).

**Conclusion:**

CDC20 may serve as a potential prognostic biomarker of human colorectal cancer.

## Background

Colorectal cancer (CRC) is one of the most commonly diagnosed cancers and, in recent years, has been the second leading cause of cancer-related death in China [1]. Surgical resection remains the primary course of action to cure CRC in patients with early clinical stages; however, even at the same stage, the recurrence and survival rates vary among patients. To date, few genomic markers, such as microsatellite instability and loss of heterozygosity at chromosome 18q, are useful for determining the prognosis of CRC. In this case, improving the molecular markers available to distinguish an unfavorable prognosis is of great importance because this group of patients may benefit from more efficient therapy.

The anaphase-promoting complex/cyclosome (APC/C) is an E3 ubiquitin ligase that can ubiquitinate certain substrates for sequential degradation through the ubiquitin proteasome pathway [2]. Recent studies have indicated that the APC/C participates in the regulation of mitosis through ubiquitinating key regulators that have distinct functions during mitosis including survivin, securin and cyclins [3,4].

The cell division cycle 20 homolog (CDC20) is a major cofactor for the APC/C through interaction with specific elements of its substrates such as the KEN-box, A-box or D-Box [5,6]. In mammalian cells, CDC20 is required for APC/C activation at metaphase and participates in mitotic exit [7]. Recently, the spindle assembly checkpoint (SAC) was found to be a monitor of the bipolar segregation of duplicated chromosomes during the metaphase to anaphase transition [2]. SAC dysfunction during mitosis leads to chromosomal instability and, thus, the generation of aneuploid cells, which are considered to be cancer cells [8]. To date, 7 components of the SAC, which are MAD1, MAD2, MAD3, BUB1, BUBR1, BUB3 and CDC20, have been identified. When an error occurs during sister chromatid segregation, the mitotic checkpoint complex (MCC) is activated, and CDC20 is sequestered by Mad2 and BubR1/Bub3, which arrests the cell cycle [8]. An abnormal level or dysfunction of CDC20 may therefore abolish mitotic arrest and thus promote premature anaphase by deregulating APC activation, resulting in aneuploidy in the daughter cells [9]. Interestingly, CDC20 was recently found to be overexpressed in many types of human cancers, including human non-small cell lung cancer, pancreatic cancer, glioma and oral cancer [10-13].

However, the clinical role and function of CDC20 in CRC development remain poorly understood. In this current study, we investigated CDC20 expression in CRC and evaluated its prognostic significance by correlating CDC20 protein expression with the clinicopathologic features and survival of patients in 244 archived CRC samples.

## Methods

### Cell culture

The human colon epithelial cell line, NCM460, was obtained from Sun Yat-sen University Cancer Center; two other human colon epithelial cell lines, CCD841-coN and CCD112-coN, were purchased from ATCC. Human colon cancer cell lines DLD1, HT29, HCT116, Lovo, SW620 and THC8307 were obtained from Sun Yat-sen University Cancer Center. NCM460, CCD841-coN and CCD112-coN cells were cultured in MEM medium supplemented with 10% fetal bovine serum (FBS). DLD1, HT29, HCT116, Lovo and THC8307 cells were cultured in RPMI 1640 medium supplemented with 10% FBS. SW620 cells were cultured in Leibovitz’s L-15 medium supplemented with 10% FBS. All cells were grown in 10-centimeter cell culture dishes (NEST Biotechnology, Wuxi, China) in 5% CO_2_ in a humidified atmosphere at 37°C.

### Tumor specimens

A total of 244 paraffin-embedded archived samples were used for immunohistochemistry, including 126 samples with adjacent non-tumorous (ANT) tissues and 20 samples with liver metastasis. All patients underwent their initial surgery between 2001 and 2009 at Sun Yat-sen University Cancer Center after providing informed consent. None of these patients received preoperative therapy. Informed consent from patients and approval from the Institute Research Ethics Committee were obtained before the use of the clinical materials. Clinical and pathologic classification and staging were determined according to the American Joint Committee on Cancer (AJCC) TNM staging system. Table 1 shows the clinical information related to the 244 CRC samples. Overall survival (OS) was defined as the interval between the date of surgery and the date of death or last known follow up.

**Table 1:** Clinicopathological characteristics and CDC20 expression of 244 patient samples of colorectal cancer

	**Number of cases (%)**
**Gender**	
Male	158 (64.8)
Female	86 (35.2)
**Age (years)**	
≤ 50	85 (34.8)
> 50	159 (65.2)
**Location**	
colon	113 (46.3)
rectal	131 (53.7)
**Clinical stage**	
I	42 (17.2)
II	53 (21.7)
III	72 (29.5)
IV	77 (31.6)
**T classification**	
T1	16 (6.6)
T2	40 (16.4)
T3	104 (42.6)
T4	84 (34.4)
**N classification**	
N0	107 (43.9)
N1	75 (30.7)
N2	62 (25.4)
**M classification**	
M0	167 (68.4)
M1	77 (31.6)
**Pathologic differentiation**	
Poor	55 (22.5)
Moderate	168 (68.9)
Well	21 (8.6)
**Vital status (at follow-up)**	
Alive	142 (58.2)
Death (All colorectal cancer-related)	102 (41.8)
**Expression of CDC20**	
Low expression	130 (53.3)
High expression	114 (46.7)

### Western blotting

CDC20 expression was compared between colon cancer cells and colon normal epithelial cells by Western blotting analysis, as described previously [14]. CDC20 protein expression was determined with anti-rabbit immunoglobulin G (1:2000; Bethyl) according to the manufacturer's suggested protocols. A rabbit anti-α-tubulin monoclonal antibody (1:20,000; Abcam) was used as the loading control.

### Immunohistochemistry (IHC)

Altered CDC20 protein expression was also studied in 244 human colorectal cancer tissues by immunohistochemistry, as described previously [15]. Briefly, the tissue sections were deparaffinized, rehydrated, endogenous-peroxide-blocked and antigen-retrieved sequentially and were then incubated with a rabbit anti-CDC20 antibody (1:150; Bethyl) overnight at 4°C. Then, the tissue sections were washed with PBS and treated with anti-rabbit secondary antibody for 20 minutes, followed by further incubation with the streptavidin horseradish peroxidase complex. The sections were developed with diaminobenzidine tetrahydrochloride (DAB) and further counterstained with hematoxylin. The degree of immunostaining was evaluated by two independent observers who were blind to the clinical data of the patients. The percent of positive cells was scored as ≤ 10% = 0, >10% to ≤ 25% = 1, >25% to ≤ 50% = 2, >50% to ≤ 75% = 3 or >75% = 4. The intensity of nuclear staining was scored as negative = 0, weak = 1, moderate = 2, or strong = 3. The two scores were then multiplied to calculate the final score. CDC20 expression was considered low if the final score was equal to or less than four; otherwise, CDC20 expression was considered high.

### Statistical analysis

All statistical analyses were carried out using the SPSS 16.0 statistical software package (SPSS Inc., Chicago, IL). CDC20 expression was compared between tumor tissues and matched ANT tissues or matched liver metastatic tissues using the Wilcoxon signed rank test. The relationship between CDC20 expression and clinicopathologic characteristics was analyzed by Pearson’s chi-squared test. Survival curves were plotted by the Kaplan-Meier method and compared using the log-rank test. Survival data were evaluated using univariate and multivariate Cox regression analyses. A *P*-value of less than 0.05 was considered statistically significant.

## Results

### Elevated CDC20 expression in colorectal cancer and metastatic liver tissues

**Figure 1:**
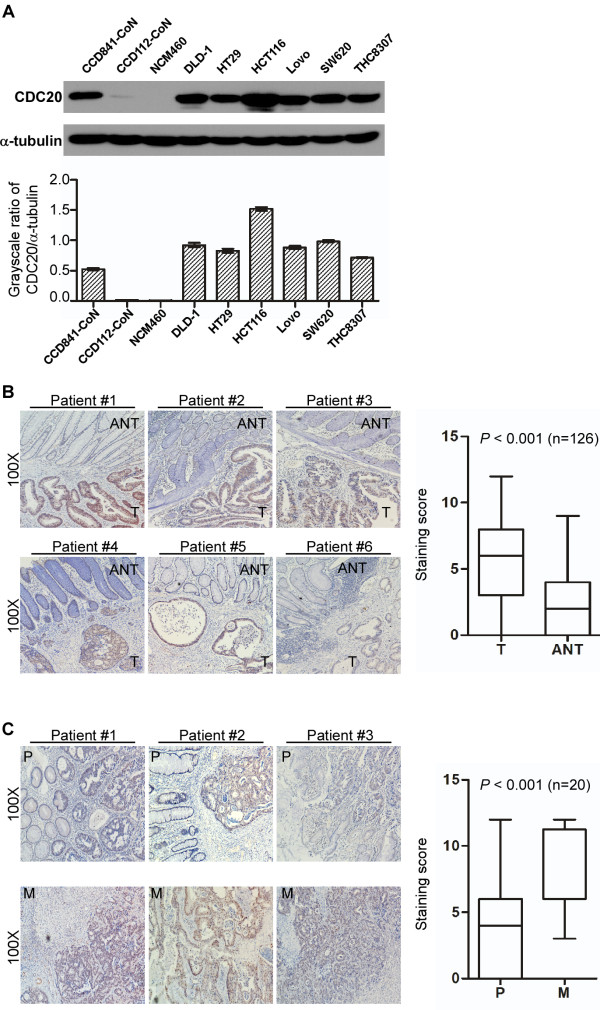
**Increased expression of CDC20 in colorectal cancer. A**, Western blotting analysis of CDC20 expression in normal colon epithelial cell lines and colon cancer cells. The quantitative data are shown. Bars: SD; *: *P* < 0.05. **B**, Immunostaining of CDC20 in six pairs of representative colorectal tumor tissues (T) with adjacent non-cancerous tissues (N) (left panel). The quantitative analysis is also shown (right panel, Wilcoxon signed rank test, n = 126, *P* < 0.001). **C**, Immunostaining of CDC20 in three pairs of representative primary tumor tissues (P) with liver metastasis tumor tissues (M) (left panel). The quantitative analysis is also shown (right panel, Wilcoxon signed rank test, n = 20, *P* < 0.001).

As shown in Figure 1, CDC20 expression was evaluated in colorectal cancer cell lines and tissues to investigate the role of CDC20 in tumorigenesis. Western blotting analysis revealed elevated CDC20 expression in colon cancer cell lines (DLD1, HT29, HCT116, Lovo, SW620 and THC8307) compared with normal colon epithelial cell lines (NCM460, CCD841-coN and CCD112-coN) (Figure 1A, upper row) (Figure 1A, lower row). CDC20 overexpression was further evaluated by IHC in 244 paraffin-embedded colon cancer tissues. As shown in Figure 1B, CDC20 was primarily localized in the cancer cell nucleus. Interestingly, a comparison of CDC20 expression in tumor tissues and ANT tissues in the paired 126 cases showed significant CDC20 overexpression in cancer cells (Figure 1B, right panel, *P* < 0.001, Wilcoxon signed rank test); the representative slides are shown in Figure 1B, left panel. Moreover, CDC20 expression was further increased in the matched metastatic liver tissues compared to the primary cancer tissues, and representative slides are shown in Figure 1C, left panel. The primary colorectal cancer tissues were compared to their respective metastatic liver tissues, and the increase was significant (Figure 1C, right panel, *P* < 0.001, Wilcoxon signed rank test). These results together suggested CDC20 overexpression in colorectal cancer.

### Correlation between CDC20 expression and clinicopathologic features of colorectal cancer

We further analyzed the link between CDC20 expression and the clinical characteristics of colorectal cancer in 244 paraffin-embedded, archival primary colorectal cancer tissues by IHC. The samples included 42 cases of clinical stage I (17.2%), 53 cases of clinical stage II (21.7%), 72 cases of clinical stage III (29.5%) and 77 cases of clinical stage IV (31.6%) colorectal cancer. As shown in Table 1, 114 of the total 244 CRC cases (46.7%) demonstrated high CDC20 expression, whereas 130 cases (53.3%) had low CDC20 expression.

**Figure 2:**
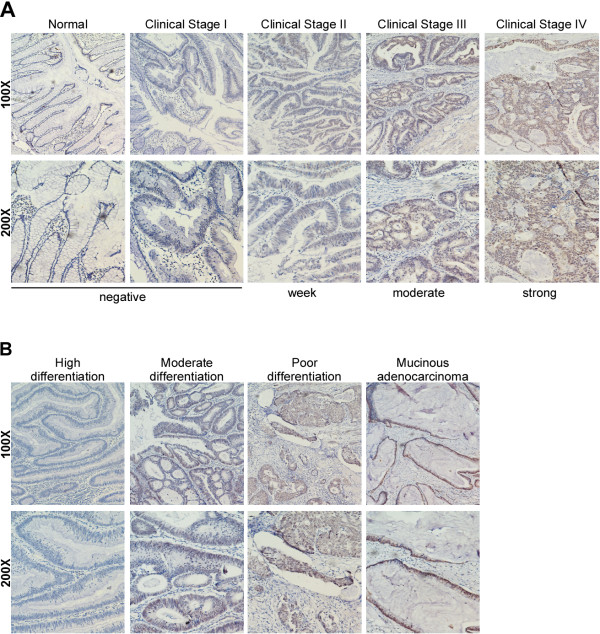
**Correlation of CDC20 expression and clinicopathologic features. A**, Increased expression of CDC20 in advanced colorectal cancer. Representative IHC slides of CDC20 expression in normal colorectal tissues/colorectal cancer tissues of different clinical stages and the staining strength are shown. **B**, CDC20 expression in colorectal cancer tissues with different levels of pathologic differentiation.

As shown in Table 2, a statistical analysis revealed a strong correlation between CDC20 expression and the clinicopathologic characteristics including clinical stage (*P* = 0.008), N classification (*P* = 0.020), M classification (*P* = 0.013), pathologic differentiation (*P* = 0.008) and vital status (*P* < 0.001). Representative IHC stained slides are presented to demonstrate the correlation between CDC20 expression and clinical stage (Figure 2A) and pathologic differentiation (Figure 2B), respectively.

**Table 2:** Correlation between CDC20 expression and the clinicopathological characteristics of colorectal cancer patients

**Characteristics**		**CDC20**		**Chi-square**
		**Low or none**	**High**	
		**No. cases (%)**	**No. cases (%)**	**test*P*-value**
Gender	Female	39 (30.0)	47 (41.2)	0.067
	Male	91 (70.0)	67 (58.8)	
Age (years)	≤ 50	43 (33.1)	42 (36.8)	0.538
	> 50	87 (66.9)	72 (63.2)	
Location	colon	61 (46.9)	52 (45.6)	0.838
	rectal	69 (53.1)	62 (54.4)	
Clinical Stage	I	26 (20.0)	16 (14.0)	**0.008**
	II	37 (28.5)	16 (14.0)	
	III	35 (26.9)	37 (32.5)	
	IV	32 (24.6)	45 (39.5)	
T classification	T1+T2	29 (22.3)	27 (23.7)	0.799
	T3+T4	101 (77.7)	87 (76.3)	
N classification	No	66 (50.8)	41 (36.0)	**0.020**
	Yes	64 (49.2)	73 (64.0)	
M classification	M0	98 (75.4)	69 (60.5)	**0.013**
	M1	32 (24.6)	45 (39.5)	
Pathologic Differentiation	Poor	21 (16.2)	34 (29.8)	**0.008**
	Moderate	93 (71.5)	75 (65.8)	
	Well	16 (12.3)	5 (4.4)	
Vital status (as followed up)	Alive	91 (70.0)	51 (44.7)	**<0.001**
	Dead	39 (30.0)	63 (55.3)	

### Association between CDC20 expression and patient survival

**Figure 3:**
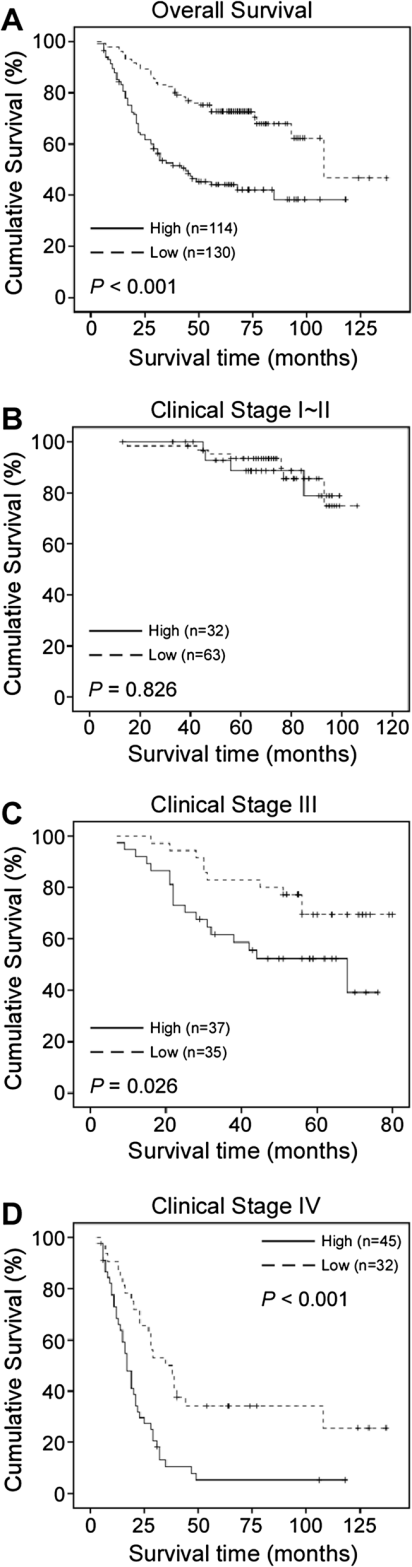
**Kaplan-Meier curves with univariate analyses (log-rank) for patients with low CDC20 expression (dotted line) versus high CDC20 expression (bold line). A**-**B**, The overall survival of patients (clinical stages I-IV (**A**) and I-II (**B**)) with low or high CDC20 expression. **C-D**, The overall survival of patients (clinical stages III (**C**) and IV (**D**)) with low or high CDC20 expression.

A Kaplan-Meier analysis and the log-rank test were carried out to evaluate the effects of CDC20 expression and clinicopathological characteristics on patient survival. Interestingly, CDC20 expression in colorectal cancer showed a negative correlation with patient survival time (*P* < 0.001). High CDC20 expression indicated a shorter overall survival (OS) (median OS: 44 months) compared to lower CDC20 expression (median OS: not reached) (Figure 3A, P < 0.001). The overall two-, three- and five-year cumulative survival rates of patients with high CDC20 expression were 61.4%, 45.8% and 27.2%, respectively. For patients with low CDC20 expression, the rates were 81.5%, 72.3% and 53.8%, respectively (Figure 3A). Furthermore, CDC20 expression was significantly correlated with OS in the advanced clinical stages (stage III and IV) (Figure 3C-3D, *P* = 0.026 (C); *P* < 0.001(D)). However, no significant correlation between OS and CDC20 expression was found in the early clinical stages (stages I and II) (Figure 3B, *P* = 0.826). Moreover, multivariate analysis indicated that N classification, M classification, pathologic differentiation and CDC20 expression were independent prognostic factors for colorectal cancer (Table 3).

**Table 3:** Univariate and multivariate analyses of various prognostic parameters in patients with colorectal cancer using Cox-regression analysis

**Variables**	**Univariate analysis**		**Multivariate analysis**		
	**No.**	***P *value**	**Hazard ratio**	**95% CI**	***P *value**
CDC20					
Low expression	130	**<0.001**	2.945	1.944-4.461	**<0.001**
High expression	114				
T classification					
T1	16	**0.014**	1.058	0.813-1.376	0.676
T2	40				
T3	104				
T4	84				
N classification					
N0	107	**<0.001**	2.036	1.536-2.699	**<0.001**
N1	75				
N2	62				
M classification					
M0	167	**<0.001**	5.733	3.641-9.028	**<0.001**
M1	77				
Differentiation					
Poor	55	0.078	0.673	0.476-0.953	**0.026**
Moderate	168				
Well	21				

## Discussion

More than one million new cases of CRC occur each year worldwide, and it has been the second leading disease-specific cause of mortality in China in recent years [1]. Genomic instability is considered a key hallmark of CRC. Briefly, there are three subtypes of genomic instability for CRC, including microsatellite instability (MSI), chromosomal instability (CIN) and CpG island methylation phenotype (CIMP) [16,17]. Studies have revealed that CIN occurs in 80%–85% of CRC and that it is the most common subtype [18]. Later studies discovered that mutations in genes that regulate the mitotic spindle checkpoint were responsible for CIN [19]. The SAC is crucial for high-fidelity mitotic chromosome segregation to maintain genome integrity. When an error is detected during sister chromatid segregation, the mitotic metaphase-to-anaphase checkpoint will be activated, inducing cell arrest. CDC20, a crucial activator of the anaphase-promoting complex/cyclosome (APC/C), is then inhibited by Mad2 and BubR1/Bub3, which prevents premature anaphase. Despite the altered CDC20 expression found in different types of cancer [10-13], the tumorigenic role of CDC20 in CRC remains unclear.

In the current study, we demonstrated for the first time that CDC20 was overexpressed in colon cancer cells compared with normal colon epithelial cells (Figure 1A). Although there was relatively high CDC20 expression in normal colon epithelial CCD841-coN cells, most likely due to individual differences, the IHC analysis confirmed the elevated expression of CDC20 protein in the 126 CRC tissues compared with matched, adjacent non-tumor tissues (Figure 1B, *P* < 0.001, Wilcoxon signed rank test, n =126). Interestingly, CDC20 expression was further increased in metastatic liver tissues (Figure 1C, *P* < 0.001, Wilcoxon signed rank test, n = 20). Higher CDC20 expression significantly correlated with advanced tumor stage, poor pathologic differentiation and unfavorable prognosis in locally advanced and advanced clinical stages (Figure 3 and Table 2). Moreover, the multivariate analysis suggested that CDC20 was a potential independent prognostic factor for survival in CRC patients (Table 3, *P* < 0.001). However, no significant correlation was observed between CDC20 expression and overall survival in stage I~II CRC, most likely due to the good prognosis in the early stages of CRC and the limited number of cases.

Recent studies have suggested that CDC20 might be a potential target for cancer therapy. Cancer cells that do not undergo apoptosis after mitotic arrest are resistant to anti-mitotic drugs [20,21]. Premature exit from mitotic arrest is considered a mechanism for escaping apoptosis [22]. Hsiao-Chun Huang *et al.* demonstrated that blocking mitotic exit downstream by CDC20 knockdown was a better strategy for killing apoptosis-resistant, slippage-prone or SAC-defective cancer cells [23]. Another study also found that treatment of cancer cells with siRNA against CDC20 successfully induced G_2_/M arrest and suppressed cell growth [24]. In our present study, we found that clinical stage IV CRC and higher CDC20 expression were significantly associated with shorter survival time (Figure 3D, *P* < 0.001), implying a potential poor response to oxaliplatin- or irinotecan-based chemotherapy.

To date, the mechanisms involved in the regulation of CDC20 are poorly understood. One potential mechanism is transcriptional downregulation of CDC20 by tumor suppressor gene p53 [24]. Moreover, a recent study found that CDC20 could be deacetylated by SIRT2, a member of the sirtuins family [25]. Further studies are required to investigate how CDC20 is regulated in cancer cells.

## Conclusions

In the present study, we found that CDC20 was overexpressed in CRC and was important for CRC tumorigenesis. CDC20 expression was highly associated with clinical stage, N classification, M classification and pathologic differentiation. Patients with higher CDC20 expression had a shorter predicted overall survival time, and CDC20 was an independent prognostic factor. These findings may have broad implications in the clinical treatment of CRC.

## Abbreviations

AJCC: American joint committee on cancer; CRC: Colorectal cancer; DAB: Diaminobenzidine tetrahydrochloride; IHC: Immunohistochemistry; ANT: Non-tumorous tissues; OS: Overall survival; APC/C: Anaphase-promoting complex/cyclosome; CDC20: Cell division cycle 20 homolog; MSI: Microsatellite instability; CIN: Chromosomal instability; CIMP: CpG island methylation phenotype; SAC: Spindle assembly checkpoint.

## Competing interests

The authors declare that they have no competing interests.

## Authors’ contributions

WWJ conceived the study, performed the IHC and drafted the manuscript. HKS performed the WB and participated in the IHC. CDL, WDS, ZDS and BL participated in the clinical data collection. ZZL and WDS performed the statistical analysis. XRH conceived the study, participated in its design and gave final approval of the version to be published. All authors read and approved the final manuscript.
